# NURR1 Impairment in Multiple Sclerosis

**DOI:** 10.3390/ijms20194858

**Published:** 2019-09-30

**Authors:** Francesca Montarolo, Serena Martire, Simona Perga, Antonio Bertolotto

**Affiliations:** 1 Neuroscience Institute Cavalieri Ottolenghi (NICO), University of Turin, Orbassano, 10043 Turin, Italy; serena.martire@gmail.com (S.M.); simona.perga@unito.it (S.P.); antonio.bertolotto@gmail.com (A.B.); 2 Neurobiology Unit, Neurology—CReSM (Regional Referring Center of Multiple Sclerosis), AOU San Luigi Gonzaga Orbassano, 10043 Turin, Italy

**Keywords:** multiple sclerosis, nuclear receptor related 1 protein NURR1, inflammation, autoimmune diseases, NF-kB, experimental autoimmune encephalomyelitis

## Abstract

The transcription factor NURR1 is a constitutively active orphan receptor belonging to the steroid hormone receptor class NR4A. Although a genetic association between NURR1 and autoimmune inflammatory diseases has never emerged from genome-wide association studies (GWAS), alterations in the expression of NURR1 have been observed in various autoimmune diseases. Specifically, its role in autoimmune inflammatory diseases is mainly related to its capability to counteract inflammation. In fact, NURR1 exerts anti-inflammatory functions inhibiting the transcription of the molecules involved in proinflammatory pathways, not only in the peripheral blood compartment, but also in the cerebral parenchyma acting in microglial cells and astrocytes. In parallel, NURR1 has been also linked to dopamine-associated brain disorders, such as Parkinson’s disease (PD) and schizophrenia, since it is involved in the development and in the maintenance of midbrain dopaminergic neurons (mDA). Considering its role in neuro- and systemic inflammatory processes, here we review the evidences supporting its contribution to multiple sclerosis (MS), a chronic inflammatory autoimmune disease affecting the central nervous system (CNS). To date, the specific role of NURR1 in MS is still debated and few authors have studied this topic. Here, we plan to clarify this issue analyzing the reported association between NURR1 and MS in human and murine model studies.

## 1. Introduction

The nuclear receptor subfamily 4 group A member 2 (NR4A2, also known as nuclear receptor related 1 protein, NURR1) is a transcription factor belonging to the steroid nuclear hormone receptor class, along with NR4A1 (also called Nur77) [1] and NR4A3 (also called Nor-1) [2]. As a nuclear receptor, NURR1 has an N-terminus domain, a highly conserved zinc-finger DNA-binding domain, and a carboxyl-terminal ligand-binding domain characterized by highly folded hydrophobic amino acid side chains [3]. Additionally, NURR1 is an orphan receptor since its activity is not regulated by ligands and its structure is locked in a constitutively active form regulated at the transcriptional and post-translational level [4].

NURR1 has been associated to several autoimmune inflammatory diseases, such as psoriasis [5], rheumatoid arthritis (RA) [6], pemphigus vulgaris (PV) [7], and multiple sclerosis (MS) [8]. Specifically, NURR1 expression is aberrantly increased in psoriasis skin [5], in macrophages of the atherosclerotic lesions [9], and in RA synovial tissues [6]. In PV, an autoimmune blistering disease of the skin, NURR1 is reduced in peripheral blood CD4+ T cells [7]. The contribution of NURR1 in the pathogenesis of inflammatory diseases is mainly related to its capability to counteract inflammation by controlling many cellular events, such as the inhibition of pathways of the proinflammatory nuclear factor kappa light chain enhancer of activated B cells (NF-kB) and of the activator protein 1 (AP-1) [10], and the maturation of regulatory T cells [11,12].

In the central nervous system (CNS), NURR1 has a well-established role in the development and in the maintenance of midbrain dopaminergic neurons (mDA), for which it is considered a promising candidate gene to be addressed by future therapeutic strategies in dopamine-associated brain disorders [13]. NURR1 is required for mDA generation, as its ablation leads to their full agenesis [14,15]. It also plays a critical role in the migration and target area innervation of differentiating mDA in the striatum [16]. In mature mDA, NURR1 regulates genes of the dopamine (DA) signaling pathway, including tyrosine hydrolase (TH), dopamine transporter 1 (DAT1), and vesicular monoamine transporter 2 (VMAT2) [14,17,18]. Furthermore, NURR1 is implicated in neuroinflammatory processes acting in microglia and astrocytes. Specifically, NURR1, recruiting the corepressor element 1 silencing transcription factor complex (CoREST), plays an anti-inflammatory role by inhibiting the expression of proinflammatory genes of the NF-kB pathway [10]. Knocking-down NURR1 with small hairpin RNA in mice leads to an increased activation of glial cells exposed to lipopolysaccharide (LPS), with subsequent production of higher levels of inflammatory cytokine-encoding mRNAs and neurotoxic effector proteins such as inducible nitric oxide synthase 2 [10]. This exaggerated inflammatory response in microglia, further amplified by astrocytes, is responsible for the death of the TH-expressing neurons, such as the mDA [10]. These studies suggest that NURR1 protects from the loss of mDA in dopamine-associated brain disorders, such as Parkinson’s disease (PD), in part by limiting the production of neurotoxic mediators by microglia and astrocytes. Accordingly, polymorphisms and mutations resulting in reduced expression of NURR1 are associated with familial and sporadic PD [19,20,21,22].

Considering the role of NURR1 in neuro- and systemic inflammatory processes, here we review the evidences supporting its contribution to MS, an inflammatory disease affecting the CNS. This issue is still debated and controversial.

## 2. NURR1 and Multiple Sclerosis

MS is a heterogeneous autoimmune chronic disease of the CNS characterized by immune-mediated inflammation with demyelination and subsequent axonal damage [23]. The aberrant lymphocyte attacks directed against the CNS components do not just destroy the myelin sheath but also affect the integrity of neuronal structures such as axons, dendrites, and synapses [24]. In detail, the activation of autoreactive CD4+ T cells directed against CNS antigens probably occurs in the periphery. After entering the CNS, they are reactivated by resident antigen presenting cells, triggering the recruitment of additional inflammatory cells (e.g., B-cells, natural killer cells, myeloid cells) to the areas of inflammation, the secretion of inflammatory mediators, and the activation of resident microglia and astrocytes, finally resulting in the myelin damage [25]. Thus, the pathological hallmark of MS is the presence of multiple focal areas of myelin loss, called plaques or lesions, disseminated throughout the CNS.

MS is the most common nontraumatic neurological disorder in young adults, affecting about one million people in the US alone [26,27]. The disease is believed to be sustained by the composite interplay of deregulated innate and adaptive immunity, genetic susceptibility, and environmental factors. MS manifests with distinct clinical phenotypes, the most frequent being relapsing-remitting MS (RRMS), characterized by episodes of neurological dysfunction that spontaneously resolve [24]. Pathologically, relapses are associated with focal demyelinating lesions in the CNS, heavily infiltrated by immune cells. In over 75% of cases, RRMS evolves into secondary progressive MS (SPMS), where patients experience irreversible accumulation of disability associated with neurodegeneration. In a small percentage of cases, a primary progressive phenotype is observed (PPMS), where irreversible and progressive neurodegeneration starts at onset [24].

To date, there is no cure for MS, but over the past years many disease-modifying therapies (DMTs) for RRMS have become available, and have expanded the therapeutic choice [28]. DMTs target the inflammatory response in the periphery. They attenuate disease activity through a long-term modulation of inflammation and the normalization of aberrant immune responses, leading to a decreased risk of relapse occurrence, lesions accumulation, and disability progression. Unfortunately, to date, there is only one licensed DMT for progressive MS [29].

**Figure 1:**
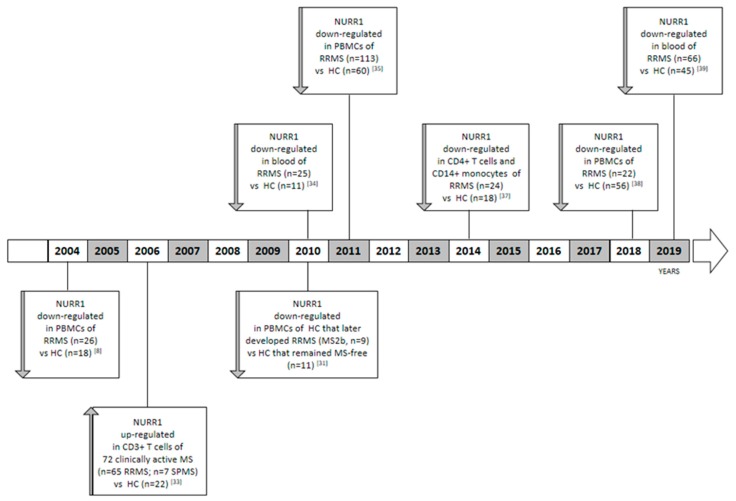
Timeline showing the published data related to the gene expression level of NURR1 in multiple sclerosis (MS).

Although a genetic association between NURR1 and MS has never been demonstrated by genome wide association studies (GWAS) [30], a relationship with the disease has been strongly highlighted by different research groups, which compared the NURR1 gene expression analysis on blood obtained from different cohorts of MS patients in comparison to healthy control cases (HC) (Figure 1). The role of the NURR1 in MS was suggested for the first time by Achiron and colleagues [31]. The authors described a blood transcriptional signature of MS, in which all the members of the NR4A family (i.e., NR4A1, NR4A2, and NR4A3) were significantly underexpressed in comparison to HC [31]. Specifically, the authors performed a microarray gene expression analysis on peripheral blood mononuclear cells (PBMCs) obtained from 26 RRMS patients and 18 HCs, including patients treated or not with immune-modulatory drugs and within the period of acute relapse or clinical remission. Due to the small sample size, the association between gene expression and clinical parameters was not evaluated. Some years later, the authors demonstrated that the downregulation of the NR4A in RRMS is not only associated to the disease state, but also precedes the clinical appearance of the disease by a period of up to nine years [31,32]. The authors analyzed the NR4A family expression on PBMCs obtained from a new cohort of HCs that later developed RRMS (MS2b, *n* = 9); and HCs that remained MS-free (*n* = 11). Obviously, in this cohort of HC subjects, nobody was subjected to immune-modulatory treatments before blood withdrawal. The most distinctive finding within the MS2b groups was the downregulation of the NR4A family gene expression level in comparison to the MS-free subjects.

On the other hand, Satoh and colleagues reported a cluster analysis in which a subgroup of MS patients showed an up-regulation of the NURR1 gene expression level in comparison to HC. Collectively, the analysis was performed on CD3+ T cells obtained from 72 clinically active Japanese MS patients, including 65 RRMS and 7 SPMS cases in comparison to 22 HC using a microarray technology. Only a sub-group of MS patients showed the NURR1 upregulation in comparison to HCs [33]. In this study, NR4A1 and NR4A3 were not analyzed. In order to explain these contrasting results, it is noteworthy that Achiron performed the analysis on PBMCs in a Caucasian population; meanwhile, Satoh used CD3+ T isolated cells obtained from a Japanese cohort of subjects. In addition, Satoh and colleagues also included SPMS patients in the MS group and not only RRMS.

To shed light on this issue, some years ago we characterized a gene expression signature of PBMCs obtained from untreated RRMS patients, in which NURR1 was significantly downregulated with respect to HCs [34]. The result emerged from the microarray analysis, and then it was validated in RT real-time PCR and for the first time at protein level in western blot [35]. We also observed that the NURR1 gene expression level negatively correlates with the aggressiveness of the pathology and clinical parameters at the time of sampling, such as the relapse rate and the Expanded Disability Status Scale (EDSS). In particular, more aggressive forms of the disease were characterized by lower levels of the NURR1 transcript [35]. Accordingly, we observed the NURR1 impairment being reverted in MS patients during pregnancy [34], a transitory state of immune tolerance, which has been described to be associated with reduced disease activity [36]. Furthermore, we reported a reduced expression of NURR1 in both CD14+ monocytes and CD4+ T cells of MS patients [37]. These data were subsequently corroborated in two independent populations of subjects [38,39], highlighting the importance of this phenomenon. Precisely, in the last ten years we measured the NURR1 gene expression level by means of RT real-time PCR analyses in five independent cohorts of subjects, always highlighting a downregulation of NURR1 in RRMS. Specifically, we enrolled 190 HC and 250 treatment naïve RRMS patients to evaluate the NURR1 gene expression, as reported in Table 1. In these studies, NR4A1 and NR4A3 were not analyzed, and patients affected by SPMS and PPMS were not included.

## 3. NURR1 and Murine Models of Multiple Sclerosis

**Figure 2:**
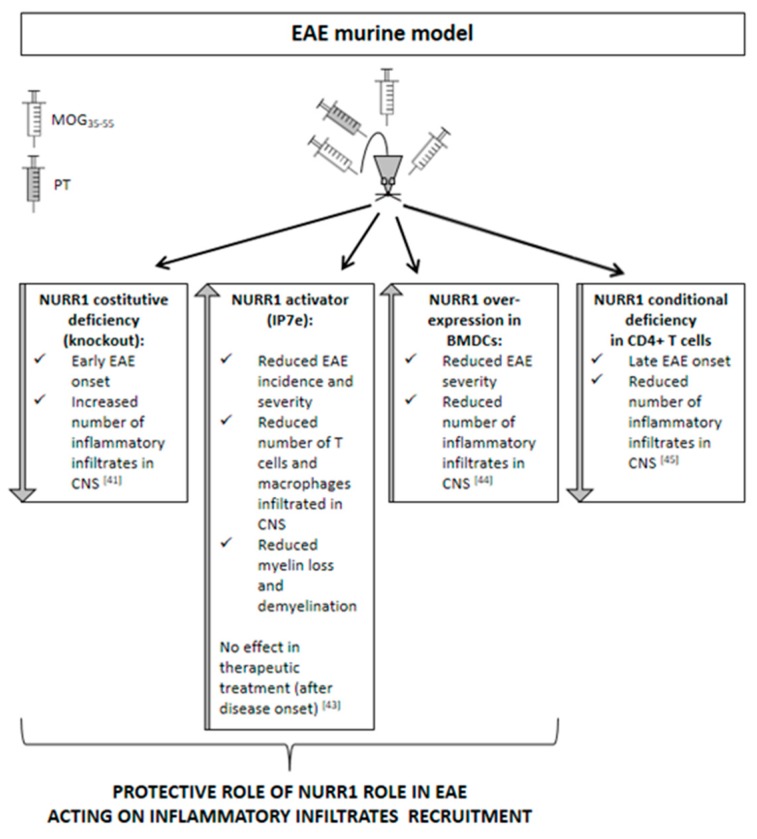
Scheamtic representation of the effects of NURR1 alteration in the experimental autoimmune encephalomyelitis (EAE) murine model.

Accordingly with the results obtained from human samples, promising data were also obtained from the murine model of MS. The most widely utilized animal model of MS is represented by the experimental autoimmune encephalomyelitis (EAE). EAE is usually induced in rodents for their versatility, relatively low maintenance cost, and genetic manipulation potential. Genetic strain and sex of the experimental animals are factors that determine the EAE phenotype that will manifest with a chronic monophasic or a relapsing–remitting profile. EAE induction can be active or passive. Active EAE is obtained via direct immunization with CNS-specific antigens, namely, highly immunogenic myelin peptides (e.g., myelin oligodendrocyte glycoprotein, MOG_35–55_; myelin binding protein, MBP_84–104_; proteolipid protein, PLP_139–151_) emulsified in complete Freund’s adjuvant (CFA) and usually complemented with the administration of pertussis toxin [40]. Passive EAE requires adoptive transfer of antigen-specific T cells obtained from actively immunized animals into recipient animals. The EAE models allow us to study the demyelinating processes with the contribution of inflammatory infiltrates that, recruited from the periphery, are responsible for the parenchymal damage. On the contrary, to investigate demyelination and remyelination in the absence of the recruitment of peripheral inflammatory infiltrates, toxin-induced demyelination models are preferred, such as the toxin-induced focal reversible demyelination model, based on the injection of lysolecithin (LPC) into the mouse corpus callosum and the cuprizone diet. Considering the function that NURR1 exerts in neuro- and systemic inflammatory processes, no studies were performed until now using animal models of demyelination without the enrollment of peripheral inflammatory infiltrates, but only the EAE model was used (Figure 2). Our laboratory found that NURR1 deficiency, obtained using the constitutive germinal knockout (KO) murine model [41], anticipates the onset of EAE induced by the MOG_35–55_ immunization. Notably, the accelerated deterioration of the EAE clinical course is related to an increased number of inflammatory infiltrates in the spinal cord of KO mice, suggesting a role of NURR1 in the recruitment of immune cells into the CNS [41]. Otherwise, Isoxazolo pyridinone 7e (IP7e) treatment, a potent activator of the NURR1-associated signaling pathway [42], delayed the EAE onset along with attenuating inflammation and neurodegeneration in spinal cords through an NF-kB-pathway-dependent process [43]. Notably, we found that the preventive treatment (i.e., started before EAE onset) with IP7e reduces the severity of EAE suppressing the accumulation in spinal cord of the cellular components necessary for the initiation of EAE, such as T lymphocytes and macrophages. Meanwhile, the therapeutic treatment (i.e., started after EAE onset) with IP7e was not able to improve the EAE outcome, supporting the suggested role of NURR1 in the acceleration of the early phases of the disease in which immune cells are recruited. In addition, we demonstrated that the IP7e is able to attenuate inflammation and neurodegeneration in spinal cords of EAE mice by an NF-kB-pathway-dependent process [43].

Recently, Saini and colleagues highlighted that NURR1 protects from EAE by promoting the differentiation of regulatory T cells, thus preventing autoimmune neuroinflammation [44]. In the experiment performed by Saini and colleagues, the bone-marrow-derived dendritic cells (BMDCs) overexpressing NURR1 were intravenously injected in MOG_35–55_-induced EAE mice. Interestingly, the mice receiving NURR1 overexpression showed less clinical severity of EAE progression and less pronounced infiltration of inflammatory cells in the spinal cord sections when compared with the control mice. In agreement with this concept, the NR4A role has been previously demonstrated in immune homeostasis [11,12]. Particularly, Sekiya and colleagues reported that the T-cell-specific NR4A-KO mice developed spontaneous systemic autoimmune disease and deficiency in regulatory T cell development [11,12].

On the contrary, the Japanese research group reported a late-onset disease and a great reduction of acute inflammation in mice lacking NURR1 in the CD4+ T cells. In this context, the NURR1 is supposed to induce the differentiation of the proinflammatory T helper 17 cells in the MOG_35–55_-induced EAE model [45]. These differences may be due to the dissimilar experimental procedures used to block or activate NURR1 expression. In fact, Raveney and co-workers used small interfering RNAs to inhibit NURR1 upregulation on the day of the EAE induction [45], while we continuously treated EAE mice with the activator of the NURR1 signaling pathway IP7e, starting when phenotypic EAE signs were not yet evident but the immunization process had already occurred [43], and we reduced the expression of NURR1 using the murine KO mouse model [41]. However, these contrasting data have never been validated by other research groups.

## 4. Conclusions

Collectively, the reported scientific literature suggests that NURR1 exerts anti-inflammatory functions not only in peripheral blood but also in target tissues, such as the skin [5], the synovial tissues [6], and the CNS [10]. Its capability make the transcription factor a promising actor during the activation of the inflammatory processes. Probably for these reasons, NURR1 is aberrantly expressed in autoimmune diseases in which inflammatory processes are altered. Indeed, the absence or the partial deficiency of NURR1 results in the exaggerated inflammatory responses characteristic of the inflammatory diseases. To date, the involvement of NURR1 in autoimmune inflammatory diseases has been highlighted but not completely dissected. In fact, NURR1 has been associated to several autoimmune inflammatory-related diseases [5,6,7], but its role in these pathological states has not yet been undoubtedly clarified.

Contrasting results have been reported in MS. To date, two independent research groups reported a downregulated gene expression level of NURR1 in hematic compartment obtained from RRMS patients in comparison to HC [8,31,32,34,35,37,38,39]. These data were obtained using different cohorts of subjects and using different techniques, such as microarray technology and RT-PCR analysis. However, only one Japanese research group highlighted the upregulation of the NURR1 gene expression level in the CD3+ T cells obtained from MS subjects, including RRMS and SPMS, with respect to HC [33]. These contrasting results could be explained considering (I) the heterogeneity of the MS disease, (II) the different techniques used to measure the expression level, and (III) mostly the choice of the biological sample used. In fact, the downregulation of NURR1 has been highlighted in whole blood [34,39], PBMCs [35,38], CD14+ monocytes, and CD4+ T cells [37], while the Japanese cohort reported the upregulation of NURR1 in the CD3+ T cells [33]. These differences make the data not entirely comparable.

To date, no results have been reported in progressive forms of MS and in the human brain parenchyma obtained from autopsy material or postmortem samples. These data could add information about the neuroinflammatory processes occurring in MS in which NURR1 seems to exert a protective role [10].

Considering that MS is a heterogeneous and widespread disorder, all these data could be better dissected in order to understand the specific contribution of NURR1 in each phase of the disease. This topic undoubtedly clarifies whether NURR1 could be considered a promising candidate gene to be addressed in future therapeutic strategies.

## Figures and Tables

**Table 1:** Cohorts of subjects enrolled to evaluate the NURR1 gene expression level.

Type of Sample	Whole Blood [34,39]		PBMCs [35,38]		CD4+ T Cells and CD14+ Monocytes [37]	
	HC	RRMS	HC	RRMS	HC	RRMS
**Sample size (N)**	11	25	60	113	18	24
	45	66	56	22		

N, number of subjects; HC, healthy controls; RRMS, treatment naïve relapsing-remitting multiple sclerosis; PBMCs, peripheral blood mononuclear cells.
